# Supplementary material for: Identification of Novel Pathways Regulated by APE1/Ref-1 in Human Retinal Endothelial Cells
Source: Int J Mol Sci. 2023 Jan 6;24(2):1101. doi: 10.3390/ijms24021101 (PMC9865623; doi:10.3390/ijms24021101)
Supplement: Supplementary file 1 [file ijms-24-01101-s001.zip › ijms-2055653-supplementary.pdf]

Identification of novel pathways regulated by APE1/Ref-1 in human retinal endothelial cells

Mahmut Mijit[1#], Sheng Liu[5#], Kamakshi Sishtla[2], Gabriella D. Hartman[2,7], Jun Wan[5,6@], Timothy W. Corson[2,3,4,6,7@], Mark R. Kelley[1,2,3,4,6,7@]

[1]Herman B Wells Center for Pediatric Research, Department of Pediatrics, Indiana University School of Medicine, Indianapolis, IN, 46202, USA
[2]Department of Ophthalmology, Eugene and Marilyn Glick Eye Institute, Indiana University School of Medicine, Indianapolis, IN, 46202, USA
[3]Department of Pharmacology and Toxicology, Indiana University School of Medicine, Indianapolis, IN, 46202, USA
[4]Department of Biochemistry and Molecular Biology, Indiana University School of Medicine, Indianapolis, IN, 46202, USA
[5]Department of Medical and Molecular Genetics, Indiana University School of Medicine, Indianapolis, IN, 46202, USA
[6]Indiana University Simon Comprehensive Cancer Center, Indiana University School of Medicine, Indianapolis, IN, 46202, USA
[7]Stark Neurosciences Research Institute, Indiana University School of Medicine, Indianapolis, IN, 46202, USA

*Correspondence should be addressed to Mark R. Kelley; mkelley@iu.edu.
#Equal contributors
@ co-senior authors

Supplementary Material

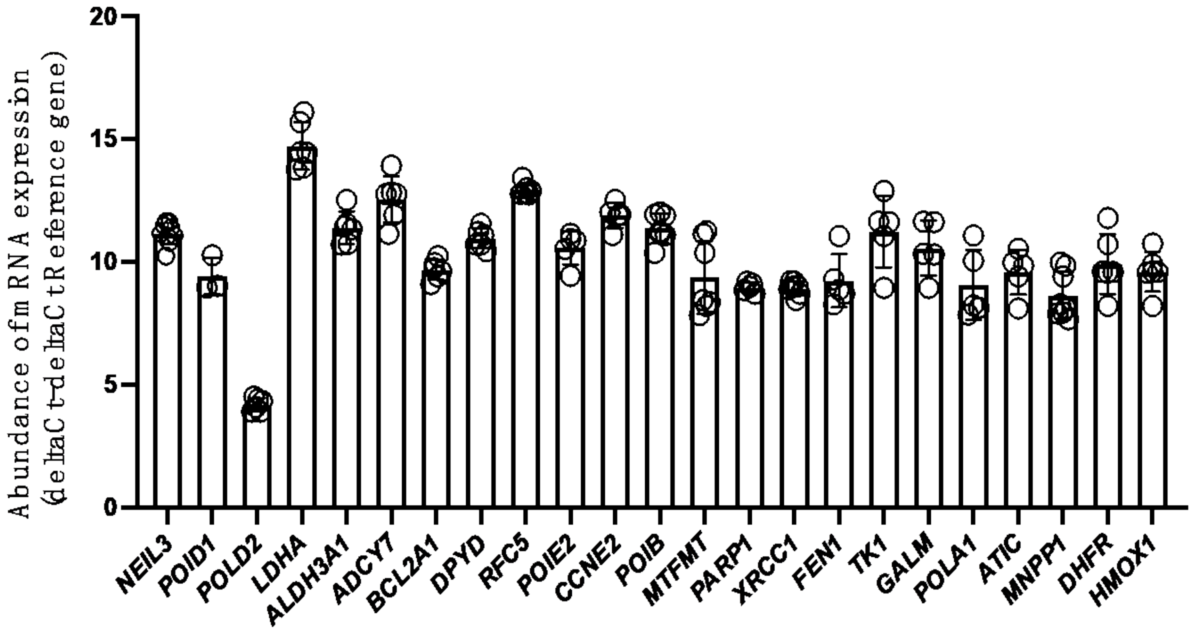

Figure S1. Relative abundance of expression levels of genes in HRECs upon basal condition. Relative mRNA expression levels of key genes from HREC RNA-seq were assessed by qRT-PCR. The data are expressed as cycle threshold (C_t value difference with respect to reference gene). *BACT* was used as reference gene. Mean ± SEM, n=3-6. At least three independent experiments were performed.

Table S1. Primer list used for qRT-PCR

Gene	Forward primer	Reverse primer
NEIL3	AGTGGTCTCCACCCAGCTGTTA	AGAGCAAGTCCTGCTTTACGGC
POLD1	ACTACACGGGAGCCACTGTCAT	GCGTGGTGTAACACAGGTTGTG
POLD2	ACTGACCCGTTCATCTTCCCAG	CAACAGCACTGTCTGGTCCTCA
LDHA	GGATCTCCAACATGGCAGCCTT	AGACGGCTTTCTCCCTCTTGCT
ALDH3A1	CTCGTCATTGGCACCTGGAACT	CTCGCCATGTTCTCACTCAGCT
ADCY7	GACGAGATGCTGTCAGCCATTG	CTCAAAGCCCTTCTCCAGGAAG
BCL2A1	GGATAAGGCAAAACGGAGGCTG	CAGTATTGCTTCAGGAGAGATAGC
DPYD	GTGGTGATGTCGTTGGTTTGGC	GTTCAGGCTTGGCAGAAACGGA
RFC5	TTCCAGTGGAGACATGCGTAGG	GTTGGCAATGTCTGACTTGAGCG
POIE2	TGCGTCCGTTTTCCTAGCAGCA	GGGCAGACATAAAGAGGTAGGG
CCNE2	CTTACGTCACTGATGGTGCTTGC	CTTGGAGAAAGAGATTTAGCCAGG
POLB	TGCAGAGTCCAGTGGTGACATG	ATGAACCTTTTGTAACTGCTCCAC
MTFMT	GCCGACTTTTGAATGAGGCTCTT	CCAGTAACTGTGTCTCCGTGAAG
PARP1	CCAAGCCAGTTCAGGACCTCAT	GGATCTGCCTTTTGCTCAGCTTC
XRCC1	CGGATGAGAACACGGACAGTGA	GAAGGCTGTGACGTATCGGATG
FEN1	ACTAAGCGGCTGGTGAAGGTCA	GCAGCATAGACTTTGCCAGCCT
TK1	AGCAGCTTCTGCACACATGACC	CTCGCAGAACTCCACGATGTCA
GALM	CAACCGAATCGCCAAAGGAACC	CGAGAACTGGACGCCATTTGAC
POLA1	GGACCAACACATCTAGCCTGGA	GGTCTGGTTTCAAAGCCATTGCC
ATIC	CCGAGAGTAAGGACACCTCCTT	GGCATCTGAGATACGCCTTTGC
MNPP1	ACAGTCGATCCAGCTGCACCTT	CTCTGCATGACCAAACTGGAGG
DHFR	CATGGTCTGGATAGTTGGTGGC	GTGTCACTTTCAAAGTCTTGCATG
HMOX1	CCAGGCAGAGAATGCTGAGTTC	AAGACTGGGCTCTCCTTGTTGC
BACT	CACCATTGGCAATGAGCGGTTC	AGGTCTTTGCGGATGTCCACGT
